# Nutritional and Nanotechnological Modulators of Microglia

**DOI:** 10.3389/fimmu.2016.00270

**Published:** 2016-07-15

**Authors:** Dusica Maysinger, Issan Zhang

**Affiliations:** ^1^Department of Pharmacology and Therapeutics, McGill University, Montreal, QC, Canada

**Keywords:** neuroinflammation, microglia, immunomodulation, nutrition, microbiota–gut–brain axis, intravital imaging, nanodelivery systems, nanomedicine

## Abstract

Microglia are the essential responders to alimentary, pharmacological, and nanotechnological immunomodulators. These neural cells play multiple roles as surveyors, sculptors, and guardians of essential parts of complex neural circuitries. Microglia can play dual roles in the central nervous system; they can be deleterious and/or protective. The immunomodulatory effects of alimentary components, gut microbiota, and nanotechnological products have been investigated in microglia at the single-cell level and *in vivo* using intravital imaging approaches, and different biochemical assays. This review highlights some of the emerging questions and topics from studies involving alimentation, microbiota, nanotechnological products, and associated problems in this area of research. Some of the advantages and limitations of *in vitro* and *in vivo* models used to study the neuromodulatory effects of these factors, as well as the merits and pitfalls of intravital imaging modalities employed are presented.

## Introduction

Neuroinflammation has been considered a detrimental factor in many neurodegenerative diseases (e.g., Alzheimer’s disease, Parkinson’s disease, and multiple sclerosis) (1–4). As the resident immune cells of the brain, microglia play a central role in neuroinflammatory processes. Traditionally, microglia were considered seminal contributors to neurodegeneration associated with neuroinflammation (5, 6). However, this view is gradually changing (7). Under normal conditions, microglia survey the brain and perform essential housekeeping functions, ranging from the scavenging of cellular debris to synaptic remodeling, but they switch from “surveyors” to “attackers” or “protectors” when challenged by pathogens, injurious stimuli, or nanoparticulates (8–10). If excessively and chronically activated, microglia exert deleterious effects in the central nervous system (CNS) by secreting proinflammatory cytokines and interfering with synaptic integrity and functions (11, 12). Microglia exhibit at least four different functions: surveillance, phagocytosis, cytotoxicity, and neuroprotection. Depending on the nature and structure of the challenger, as well as the intensity, duration, and location of the challenge, activated microglia can take on a protective or destructive role (13). Signals from healthy and damaged neurons, astrocytes, and factors from the periphery also modulate the phenotype of activated microglia (14–16). Neuroprotection is achieved through different modes of their action, e.g., (i) synaptic stripping in development and motoneuron regeneration (5), (ii) promotion of neurogenesis in the injured CNS (17, 18), (iii) phagocytosis of misfolded proteins and damaged organelles (19, 20), and (iv) production of anti-inflammatory mediators, such as interleukin-4, interleukin-10, and transforming growth factor beta (15, 21–24). Cytokines, chemokines, neurotrophins, reactive oxygen species, and glutamate are endogenous signal molecules exchanged between neurons and glia cells (25–28) that can be modulated by pharmacological agents, but the access of these agents to the CNS may be limited by the blood–brain barrier (29, 30). More recently, it was shown that the microbiome can affect the integrity and function of the blood–brain barrier, as well as the maturation and phenotype of microglia (31–34). The emergence of drug nanocarriers and nanotechnological probes has facilitated the entry of therapeutics into the brain, but some of them exerted immunogenicity. The present review will focus on key neural factors and pharmacological targets in neuroinflammation, and discuss the potential of alimentary and nanotechnological agents in modulating immune processes in the brain. The merits and pitfalls of *in vitro* and *in vivo* models of neuroinflammation will be summarized, and the use of intravital imaging modalities to investigate neuroinflammation will be reviewed.

## Modulators of Neuroinflammation

### Alimentary and Environmental Neuroimmunomodulators

Numerous genetic, environmental, and alimentary components can modulate neuroinflammation (35–37). For example, polluted urban air contains toxins, droplets, and particulates that are inhaled and travel though the blood stream, olfactory, and lymphatic systems to the brain, where they have been implicated in diseases of the CNS (37). Our daily diet can also affect neural cells, thereby altering their functions (38, 39). Rats fed high-fat diets were shown to have increased pro-inflammatory cytokines, such as tumor necrosis factor alpha (TNF-α), interleukin-6 (IL-6), and interleukin-1 beta (IL-1β), in the hypothalamus, compared to controls fed regular chow (40). The arcuate nucleus in the mediobasal hypothalamus is particularly sensitive to metabolic factors from the periphery, as it is located near the median eminence, a circumventricular organ unprotected by the blood–brain barrier (41). The activation of microglia in the arcuate nucleus of animals on high-fat diets, thus, demonstrates the direct impact of nutrition on neuroinflammation. Dietary components have also been used for therapeutic purposes as neuroprotective agents. For instance, vitamins E, C, and B have been shown to reduce oxidative stress in the brain (42, 43). The ketogenic diet is an established treatment for childhood epilepsy (44–46). It is thought that the increase in circulating polyunsaturated fatty acids can modulate ion channels, and that inflammation is altered by increasing circulating beta-hydroxybutyrate and activating hydroxy-carboxylic acid receptor 2 in immune cells (47–49). Ketogenic diets have since been proposed in neurological conditions, such as Alzheimer’s disease and brain malignancies, but further clinical studies are required to confirm these findings and explain the beneficial effects at the molecular level (50–53). The polyunsaturated fatty acid docosahexaenoic acid (DHA) is a major component of neuronal cell membranes that is metabolized into resolvins and protectins, two families of neuroprotective lipid-derived mediators (54–56). Dietary DHA was shown to attenuate ischemic brain injury and pro-inflammatory markers in animal models (57–59). We have investigated the direct effects of DHA on synaptic integrity and indirect effects *via* microglia in the hippocampal CA1 region. Our studies have shown that DHA exerts neuroprotective effects in organotypic hippocampal tissue slices by preventing post synaptic spine deterioration (59). We also showed that DHA in microglia attenuates LPS-induced inflammation through the remodeling of lipid bodies and associated organelles (60). Furthermore, Bailey et al. provided evidence for the antioxidant role of lipid bodies in glia cells and neural stem cells (61, 62).

In addition to polyunsaturated fatty acids, such as DHA, numerous endogenous and exogenous fatty acids with different degree of saturation and chain lengths have been investigated in models of physiological and pathological conditions. The gut microbiota is an important source of small chain fatty acids (SCFA). Its population is heavily influenced by diet, and in turn, it modulates both the intestinal environment and overall human health (63–65). Once absorbed, SCFA directly impact on energy homeostasis in the liver, muscles, and adipose tissues, thereby affecting obesity and insulin resistance (66). SCFA can also affect the CNS by modulating neuroendocrine and cognitive responses, particularly when changes in the gut microbiome lead to increased intestinal permeability (34, 67, 68). Emerging research on the gut–brain axis has shown that there is a tight link between the gut microbiota and the function of neural cells. The gut microbiota are necessary for the early and normal development of the brain, and contribute in programing the hypothalamic–pituitary–adrenal axis (69). In germ-free mice, microglia were found to have an immature phenotype, resulting in altered immune responses (31). Chronic enteric infections and antibiotics can also drastically modify the gut microbiome, resulting in neuropsychological symptoms (34, 70). The term “psychobiotics” has since been coined, referring to probiotics benefiting psychiatric illness, but further clinical studies are required to demonstrate the therapeutic benefits (71). While the composition and function of the gut microbiota can be affected by alimentary components, they can also be influenced by food contaminants, including nanoparticulate matter.

### Nanotechnological Immunomodulators

Mammals have been exposed to airborne, waterborne, foodborne, and other nanomaterials in the environment for millennia and have developed mechanisms to deal with them (72, 73). Nevertheless, the explosion of nanoparticles in electronics, medical devices, paints, clothing, and cosmetics raised the awareness of the nanostructured materials in everyday life, requiring careful monitoring and analysis of the level and type of nanoparticles in soil, water, and air (74). In recent years, many nanomaterials have been designed for the development of diagnostics, delivery of therapeutic agents, and implants for the replacement of missing or impaired organ parts (e.g., joints, heart) (75–78). Some of these materials are well tolerated and efficiently eliminated, but others induce immune reactions and are toxic. Nanostructured materials are mainly recognized by cells of the immune system, primarily the mononuclear phagocytic system (MPS) (79). For example, internalized carbon nanotubes can be partly degraded in macrophages and the extent of biodegradation may be a major determinant in the severity of the associated inflammatory responses (80). Nanomaterial accumulation in macrophages within clearance organs (e.g., liver, kidneys, and spleen) can initiate both acute and chronic inflammation (81, 82). Although nanomaterials can cause toxic responses in these organs, technological manipulations of their morphologies, surfaces, sizes, charges, and porosities can minimize adverse effects (83–85). The structure–activity relationship of several classes of nanoparticles and outcome measures in immune and non-immune cells has been previously discussed (86, 87).

Our laboratory is particularly interested in investigating the effects of nanomaterials on microglia because of increasing evidence that (1) microglia are the major “consumers” of nanoparticles in the CNS (10, 88, 89), (2) microglia and macrophages contribute to the maintenance and progression of glioblastoma, one of the most complex and deadly brain tumors (90), and (3) there is a structural and functional link between the CNS and lymphatic vessels (91). The discovery that lymphatic vessels lining dural sinuses are gateways between the systemic lymphatic system and the brain has recently re-defined our understanding of the immune system of the brain and is seminal in investigating neuroinflammatory and neurodegenerative disorders associated with impairments of the immune system. The majority of the studies showing either positive or negative effects of nanomaterials on the immune system focused on peripheral macrophages. This is understandable considering that most foreign materials are taken up by these cells. However, brain cancers, such as gliomas, are infiltrated mainly by the brain macrophages, the microglia. In fact, the proportion of microglia in low-grade gliomas can exceed (>35%) the normal microglia contribution (10–15%) in non-neoplastic brains. The majority of non-neoplastic cells in gliomas are tumor-associated macrophages (TAM) either originating from the periphery or intrinsic to the brain (90, 92). These cells form the microenvironment of the brain tumor and play a major role in the maintenance and progression of the cancer cells. They can contribute to cancer survival, invasiveness, and proliferation. Although the mechanisms underlying microglia stimulation of low- and high-grade gliomas are not fully understood, the existence of a unique tumor microenvironment resulting from the infiltration of central and peripheral macrophages provides an opportunity to establish more effective chemotherapeutic interventions (93). Achieving this goal is not simple because of the considerable diversity and plasticity of macrophages and microglia. The common classification of M1 polarization, deemed pro-inflammatory, and M2, designating alternatively activated macrophages (with subclasses M2a, M2b, and M2c), seems inadequate for TAM. RNA microarray analyses indicated that about 1000 transcripts were found to be differently expressed in glioblastoma-associated microglia and macrophages relative to control microglia. The expression patterns only partially (<50%) overlapped with reported gene signatures for M1 and M2 macrophages (94). Therapeutic interventions targeting glioblastoma cells alone usually failed because of the contribution of the complex environment made of surrounding cells and brain tumor stem cells (95, 96). The problem is that macrophages and microglia secrete growth- and invasion-promoting factors, whereas brain tumor stem cells residing in perivascular niches often give raise to the resistance to radiation and chemotherapy (97–100). By contrast, some data suggest that the ketogenic diet combined with standard cancer treatment could increase the sensitivity of cancer cells toward therapies due to their reliance on glycolytic metabolism (101). Such a diet could also decrease inflammation caused by infiltrating macrophages and microglia. Although the results are encouraging, additional clinical trials are required to confirm the previous findings, suggesting the beneficial effects of the ketogenic diet (102). Immunomodulation of the glioma microenvironment by nanoparticles is also an attractive therapeutic avenue to reduce tumor invasiveness and growth. Data from preclinical and clinical studies are encouraging despite limitations and hurdles, which need to be overcome before this strategy becomes more widely applied (103–105).

**Figure 1:**
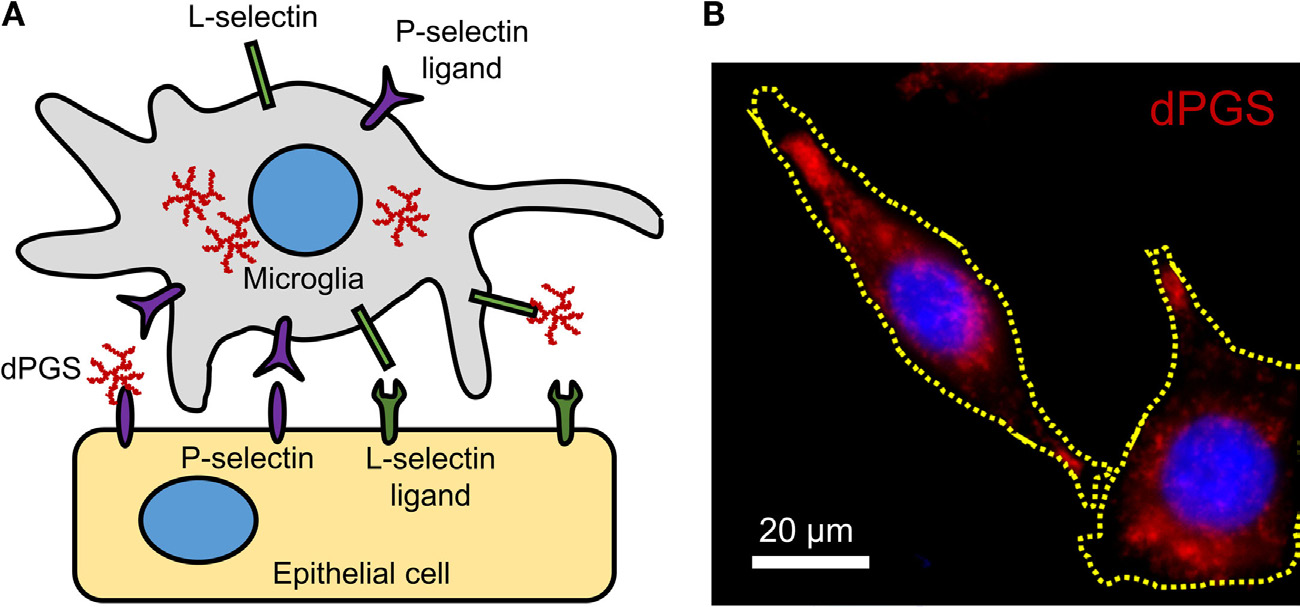
**(A)** Molecular mechanism of dPGS binding to L-selectins and P-selectin ligands. **(B)** Fluorescence micrograph of fluorescently labeled dPGS (red) in microglia. Nuclei are labeled with Hoechst 33342 (blue).

In inflammation, immunomodulation using nanoparticles could provide suitable alternatives to standard treatment strategies because of the versatility of particle surface modifications, compositions, and charges. Particles with a negative surface charge can bind to monocytes, marking them for sequestration by the spleen and preventing their migration and participation at the inflammation site (65). Interesting examples of polyanions with anti-inflammatory effects are dendritic polyglycerol sulfates (dPGS) (106–108). Studies with dPGS suggest that they are effective anti-inflammatory agents *per se* with strong inhibitory effects on inflammation-induced degenerative changes in microglia and the ability to rescue dendritic spine morphology (108). Their L-selectin binding in the low nanomolar range, limited impact on blood coagulation, and minor activation of the complement system render them attractive anti-inflammatory agents (106). A simplified molecular mechanism of dPGS binding to selectins and their intracellular location in microglia is illustrated (Figure 1).

Mechanisms for nanoparticle-induced tolerance and reduction of inflammation severity have been previously reviewed in Ref. (109). Although there are still numerous unanswered questions related to the mechanisms of nanoparticle–immune system interactions, it is anticipated that in the next decade, clinical studies will show if negatively charged biodegradable nanoparticles (e.g., polylactic–polyglycolic acid) will reduce severe inflammations in myocardial infarction and acute encephalitis syndrome. If these and similar studies show a positive outcome, nanoparticle-based therapies could become a valuable addition to existing therapies targeting the immune system (110). However, a series of safety testing and validation has to be performed in preclinical and clinical investigations. A tiered approach for assessing nanoparticle compatibility with the immune system *in vitro* during the early phase of preclinical development, strategies for designing early phase preclinical immunotoxicity screening, and challenges associated with these investigations have been reviewed in Ref. (86). Despite disappointments due to the lack of standards and standardized procedures, limited understanding of underlying mechanisms involved in nanoparticle–immune cell interactions, inadequate nanoparticle characterization and incomplete knowledge about plasma proteins and their interactions with nanoparticle surfaces under physiological and pathological conditions, results obtained so far have provided a baseline for investigations to harness biocompatible and safe nanomaterials for immunomodulation.

## Models of Neuroinflammation

### *In Vitro* Models

**Figure 2:**
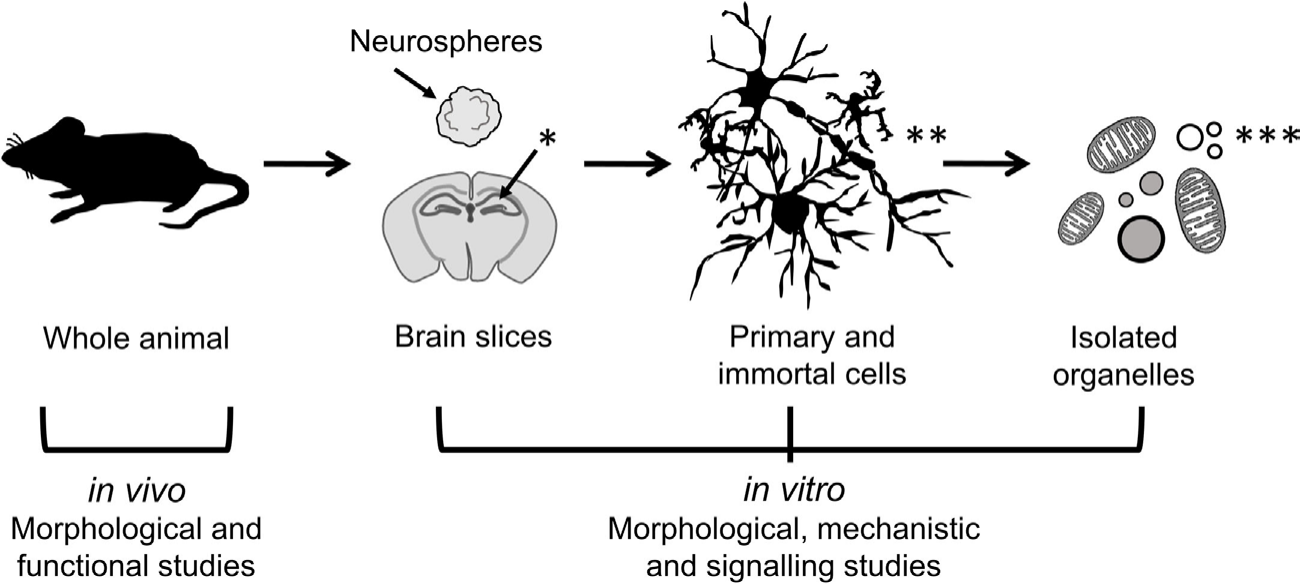
**Models of different complexity used to study the effects of immunomodulators in neural cells**. *In vivo* models of neuroinflammation are most suitable for morphological and functional studies, while *in vitro* models of neural cells in 2D (primary and immortal dissociated cells) and 3D (neurospheres and brain slice cultures) are useful for morphological, mechanistic, and signaling studies. Isolated organelles can be used to investigate mechanisms of inflammation at the subcellular level. [*Hippocampus (hippos = horse; campos = sea monster); **neurons, microglia, astrocytes; ***organelles: mitochondria, lipid droplets, lysosomes, nucleoli.]

Neuroinflammation involves complex intercellular communication between different neural cell types organized into intricate networks. Thus, suitable primary neural cells in 3D cultures (prepared either from dissociated cells or organotypic slices) are preferable to cell line models grown in 2D (Figure 2). However, both types of *in vitro* models have important limitations (Table 1). Phenotypic traits of primary cells are often lost following *in vitro* culture, particularly in monolayer and monocultures. Microglia are ramified in the healthy brain and in astrocyte co-cultures, but in the absence of astrocytic support, they take on various morphologies (e.g., amoeboid, spindle, and rod like) (111). Astrocyte-conditioned media are only partly effective in maintaining the ramified morphology of microglia, because astrocytes provide not only soluble (e.g., granulocyte macrophage colony-stimulating factor and colony-stimulating factor 1) but also non-diffusible factors. An astrocyte feeder layer is commonly used to support microglia and neuronal cultures alike (112, 113). This can be achieved using a two-chamber culture system comprising an enriched microglia culture separated from an enriched astrocyte culture by an inset with a porous membrane.

**Table 1:** **Models of immunomodulation: from *in vitro* to *in vivo* models**.

	Inflammatory stimuli	Endpoints	Advantages (+) and limitations (−)	Reference
***In vitro* models (2D)**				
Immortalized cells lines Primary dissociated cells	– Bacterial toxins (e.g., LPS) – Pro-inflammatory cytokines (e.g., TNFα) – Protein aggregates (e.g., amyloid-β) – Environmental pollutants (e.g., heavy metals) – Organic and inorganic nanocrystals (e.g., cholesterol, quantum dots)	– Released soluble factors (e.g., cytokines, chemokines) – Protein expression and enzyme activity (e.g., caspase-1) – Phagocytosis – Morphology and function of organelles (e.g., mitochondria, lysosomes) – Cell death (e.g., pyroptosis, apoptosis)	(+) Homogeneous cell population (−) Abnormal cell biology	(114–116)
			(+) Non-cancerous cells (+) Cells can be isolated from specific brain regions (−) Finite retention of phenotypic traits	
***In vitro* models (3D)**				
Organotypic brain slices Acute brain preparations	– Bacterial toxins (e.g., LPS) – Pro-inflammatory cytokines (e.g., TNFα) – Protein aggregates (e.g., amyloid-β) – Environmental pollutants (e.g., heavy metals) – Organic and inorganic nanocrystals (e.g., cholesterol, quantum dots) – Physical injuries – (e.g., “wound in the dish”)	– Released soluble factors (e.g., cytokines, chemokines) – Protein expression and enzyme activity (e.g., caspase-1) – Morphological and functional properties of neurons – Cell death (e.g., pyroptosis, apoptosis)	(+) Useful to study neurogenesis and neural development (−) Finite retention of neurogenic properties	(117–119)
			(+) Preserved brain structure and cell population (−) Damage from slicing can alter the maturation of neuronal circuitry	(120–123)
			(+) Neuronal circuitry close to *in vivo* conditions (+) Cultures can be derived from donors of any age (−) Damage from slicing can interfere with experiments	(124–126)
***In vivo* models**				
Wild-type animals	– Bacterial toxins (e.g., LPS) – Pro-inflammatory cytokines (e.g., TNFα) – Protein aggregates (e.g., amyloid-β) – Environmental pollutants (e.g., heavy metals) – Physical injuries (e.g., stroke, traumatic brain injury)	– Released soluble factors (e.g., cytokines, chemokines) – Protein expression and enzyme activity (e.g., caspase-1) – Circuit integrity – Cognitive and physical performance – Clinical signs of pain and distress, weight and survival	(+) Complete, normal biological system (+) Useful to study cognitive and physical functions (−) Variability between animals (−) Higher cost and logistic requirements	(1, 127–130)
Transgenic animals Knock-in Knock-out Optogenetic		– Released soluble factors (e.g., cytokines, chemokines) – Protein expression and enzyme activity (e.g., caspase-1) – Circuit integrity – Cognitive and physical performance – Clinical signs of pain and distress, weight and survival – Tracking of bioluminescent or fluorescent tags	(+) Complete, normal biological system (+) Possible to study cognitive and physical functions (−) Variability between animals (−) Higher cost and logistic requirements (−) Off-target effects and mosaicism (−) Breeding problems and lower survival rates	

Immortalized microglia cell lines were initially established from rodents in the 1980s, and the first human cell line was reported in 1995 (114). N9 and BV2 are among the oldest and best-described murine microglia cell lines, while CHME and HMO6 are the main human microglia cell lines. Recently, another immortalized microglia cell line was generated from the adult murine brain (131). Beside the practical advantages of an established cell line, immortalized microglia provide a relatively homogeneous cell population that retains the phagocytic and secretory abilities of their primary counterparts. However, surface markers vary from cell line to cell line, and as with any continuous cell culture, phenotypic traits may change as cells differentiate over time (114). The systematic analysis of primary mouse and human microglia genes and microRNAs identified a unique molecular signature that was distinct from peripheral immune cells and immortalized microglia cell lines. This striking difference between primary and immortalized cells indicates that continuous cell lines are not always suitable to answer some questions, such as the role of surface markers highly expressed in human or mouse microglia [e.g., purinergic receptor P2Y, G-protein coupled, 12 (P2ry12) in human, and Fc receptor-like S (FCRLS) in mouse microglia] (132, 133).

Brain slices are 3D, *ex vivo* models with partial brain architecture and synaptic circuitries. These models are used to investigate intercellular communication between neural cells under “physiological” and pathological conditions. Organotypic brain cultures are usually prepared from postnatal animals (days 3–9), and slices are maintained in culture until the maturation of the synaptic networks. Although the structural development of organotypic brain slices has been found to be largely comparable to that of age-matched animals, it has been reported that these *ex vivo* cultures had increased dendrite numbers and glutamatergic synaptic currents resulting from the rewiring of axons damaged during the initial slice preparation (120). Nevertheless, the preservation of tissue structure and the presence of microglia in organotypic brain slices are major advantages in the study of neuroinflammation. Acute brain slices are similar to organotypic brain cultures. They can be harvested from animals of any age, and experiments are typically completed within hours. However, the biomechanical stress caused by tissue slicing, presence of damaged cells, and release of soluble factors from these cells must be considered when interpreting results from such preparations (134).

**Figure 3:**
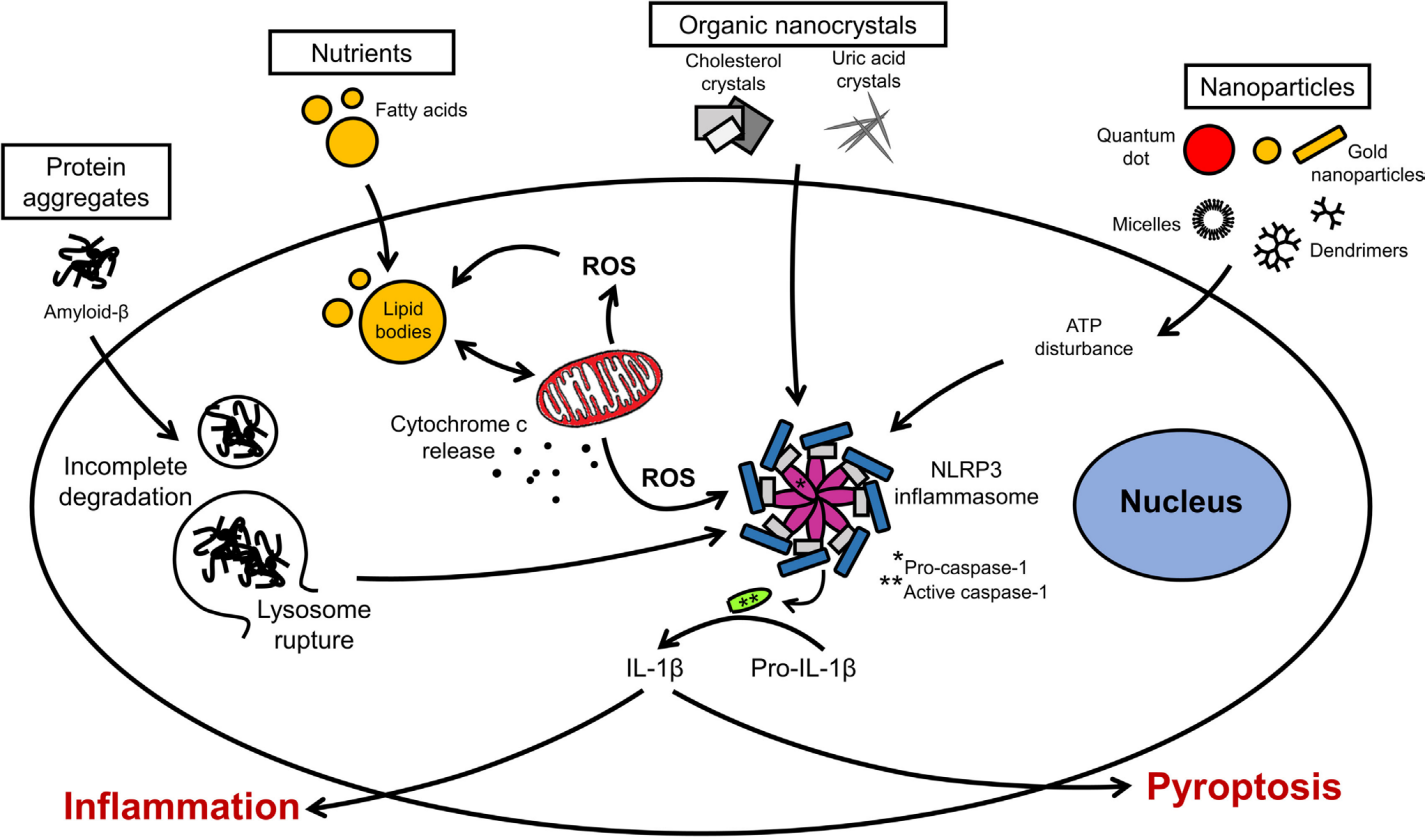
**Organellar remodeling in inflammation**. Multiple pro-inflammatory stimuli can disrupt redox homeostasis in microglia. Mitochondria are the major source of reactive oxygen species (ROS). Excessive ROS induces the formation of lipid bodies and impairs their communication with intracellular organelles. Several signal transduction pathways implicated in inflammation converge on the inflammasome. Inflammasome activation leads to the caspase activation and cytokine release. Modulation of these pathways can lead to resolution of inflammation or exacerbation with pyroptotic cell death.

Inflammation in neural cells can be induced using pathogen-derived ligands, pro-inflammatory cytokines, and injurious stimuli. Among the most common pro-inflammatory stimuli is lipopolysaccharide (LPS), an endotoxin from Gram-negative bacteria, which binds to toll-like receptor 4 (TLR4) on microglia, astrocytes, oligodendrocytes, and neurons (14, 135). The production of cytokines (e.g., interleukin-1 beta, interleukin-6, interleukin-18, interleukin-33) by microglia in response to LPS is mediated by the inflammasome, a multiprotein complex typically composed of pro-caspase-1, the adaptor molecule apoptosis-associated speck-like protein containing a caspase recruitment domain (ASC) and nucleotide-binding oligomerization domain, leucine-rich repeat-containing receptor (NLR) family proteins (Figure 3) (136). Different types of inflammasomes can assemble depending on the nature and intensity of the stimulus, and many members of the NLR family can facilitate the assembly (e.g., NLRP1, NLRP7, and NLRP12). In particular, the NLR family, pyrin domain-containing 3 (NLRP3) inflammasome is common in neuroinflammation-associated disorders, and can be regulated by a wide variety of factors, such as pathogen-associated molecular patterns, damage-associated molecular patterns, COX-2 activity, and damaged mitochondria (137–139). In Alzheimer’s disease, traumatic brain injuries (TBI), and MS, the NLRP3 inflammasome was found to exacerbate inflammatory responses and damage mediated by microglia (140–143). Notably, hyperactivation of microglia characterized by inflammasome activation and cytokine release can lead to the programed cell death pyroptosis in neural cells (144–146). Pro-inflammatory cytokines are major inducers of immune activation, both in the peripheral and central immune systems. These include, among others, TNF-α, IFNγ, IL-1β, and IL-6 (26, 147, 148). Modulation of IL-6 classical and trans-signaling has been exploited for therapeutic interventions in several preclinical and clinical trials (149, 150). The evolutionary conserved glycoprotein 130 (gp130) system inspired the development of sgp130Fc, an effective pharmacological tool to distinguish classical from trans-signaling. The results from phase III studies with sgp130Fc are awaited – it is anticipated that blockade of trans-signaling will prove to be superior to the global blockade of IL-6 signaling by the neutralizing antibody tocilizumab. Recent studies showed that the small molecule LMT-28 can also block trans-signaling of IL-6 (151). LMT-28 is stable, simple to synthesize, and functions by binding directly to gp130. Clinical data for its effectiveness in neurological disorders are not yet available. Anti-inflammatory cytokines, such as interleukin-4 and -10, can dampen the effects of pro-inflammatory stimuli. The production of these secreted factors can be monitored using enzyme-linked immunosorbent assays (ELISA). Inflammation induced by ischemic and TBI is difficult to replicate *in vitro*, but some morphological and biochemical changes can be assessed in simplified models. For instance, oxygen and glucose deprivation (OGD) is often used to mimic brain ischemia and induce the activation of toll-like receptors 2 and 4 in primary cortical neurons (135). Transection, compression, hydrostatic pressure, and stretch injuries are other examples of brain “injuries in the dish” (134).

### *In Vivo* Models

**Figure 4:**
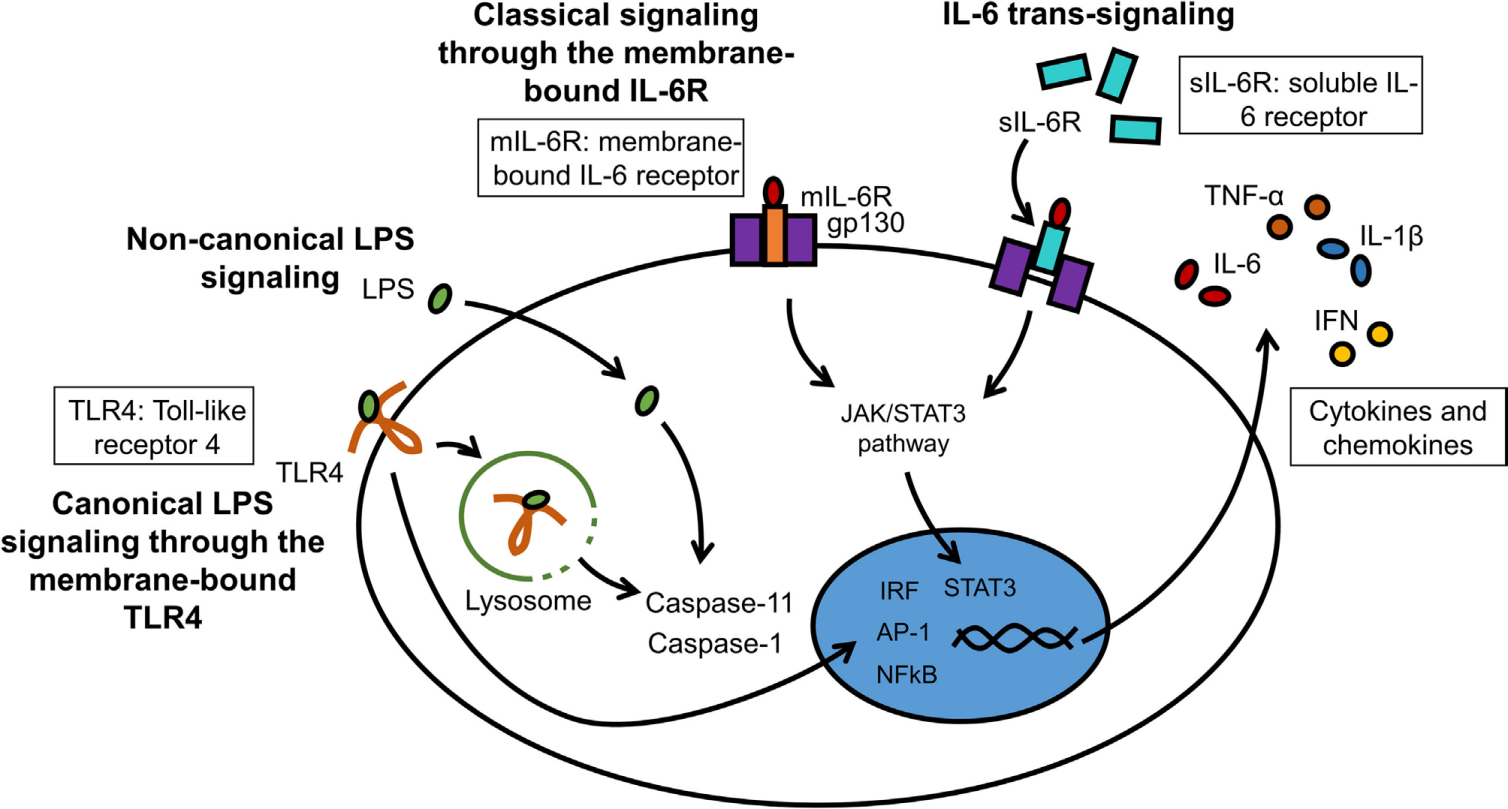
**LPS and IL-6 signaling in microglia**. LPS can interact with membrane-bound TLR4 (canonical signaling) or can enter the cytosol independently from TLR4 (non-canonical signaling). The major cytosolic receptors for LPS are pro-inflammatory caspases. IL-6 binds either to the membrane receptor IL-6R (mIL-6R; classical signaling) or to the soluble IL-6 receptor (sIL-6R; trans-signaling). These receptor complexes subsequently bind to gp130 to initiate intracellular signaling cascades.

A great number of animal models of neuroinflammation are available today, many of which are disease specific (see examples in Table 1). Although transgenic animals are popular to examine the effects of gene knock-in and knock-out, wild-type animals remain necessary to understand the fundamental pathophysiology of neuroinflammation. LPS can be injected either systemically or intracranially. Circulating LPS rapidly causes an inflammatory response in the brain, first at the circumventricular organs, then across the CNS (152). Although the choice of LPS serotype has little impact on TLR4 stimulation, it can significantly affect *in vivo* studies involving the adaptive immune system. The degree of purity of the LPS is also an important factor, as products of lesser quality can contain other pathogen-associated molecules that will alter the potency of the LPS and the magnitude of the inflammatory response. Systemic injection is often administered intraperitoneally, intravenously, or by stereotaxic administration directly into the brain parenchyma. The stereotaxic apparatus holds the head of the animal in place and a stereotaxic atlas is used to determine the coordinates for the site at which a small hole in the skull should be drilled to access a specific site in the brain (153). Until recently, innate recognition of LPS was limited to its membrane receptor TLR4/MD-2-stimulated cytokine transcription. Therapeutic intervention by Eritoran has achieved very moderate success in sepsis (154). This could be in part because of the existence of non-canonical LPS signaling induced by cytosolic LPS. This non-canonical signaling via intracellular LPS activates pro-inflammatory caspases – caspase-11 in mice and caspase-4/5 in humans – and does not depend on TLR4 (155–157). LPS binding to caspases induces their oligomerization, which is a prerequisite for caspase activation. A simplified model of canonical and non-canonical signaling by LPS is illustrated (Figure 4). Resulting CNS complications, such as encephalopathy, are mainly mediated by neuroinflammation and oxidative stress (158). Aside from LPS administration, inflammation can be induced more globally by bacterial infections. A standard method to induce polymicrobial sepsis is cecal ligation (159). It is easily performed, and the severity of the disease can be controlled to a certain extent (160). However, there is a high mortality rate, and variable outcomes have been observed between animals and laboratories (161).

Inflammatory processes in transgenic models of neuroinflammation often result indirectly from the expression of a disease-specific mutant gene, and most models were developed for the study of neurodegenerative diseases. The APP/PS1 mouse model, for instance, is used in the study of Alzheimer’s disease. These mice express a chimeric amyloid precursor protein and a mutant human presenilin-1, causing the accumulation of amyloid-beta plaques by the age of 6 months, extensive neuroinflammation and, later on, memory impairment (162). By contrast, it was recently suggested that the amyloid beta peptide can protect against microbial infection in a mouse model of Alzheimer’s disease (163). This is an intriguing proposition, raising the possibility that amyloid beta may play a protective role in innate immunity through its binding to microbial cell walls via heparin-binding domains. In the adeno-associated virus-alpha-synuclein mouse model of Parkinson’s disease, the animal expresses alpha-synuclein under the control of a viral promoter. This results in the loss of dopaminergic neurons, as well as the activation of microglia (164). For the study of amyotrophic lateral sclerosis, transgenic mice expressing a mutant superoxide dismutase 1 gene were observed to show astrocyte and microglia activation, leading to motoneuron degeneration and muscle atrophy (165, 166). Transgenic mouse models used to investigate neuroinflammation can provide valuable information on morphological, biochemical, and functional changes in neural cells, but they have limitations that must be considered in the context of human pathology (1). Other knock-out and knock-in animals have also been employed to study the role of key mediators of neuroinflammation. Caspase-1 knock-out mice, for example, seemed more resistant to ischemia-induced neural cell death than wild-type animals (167). More recently, the clustered regularly interspaced short palindromic repeats (CRISPR) and CRISPR-associated protein-9 (Cas9) gene editing technique has generated considerable excitement, as it was successful in targeting single or multiple genes in the mouse brain (168). The technique allows the generation of mutant animals with ease and efficiency compared to the traditional transfection of mouse embryonic stem cells. However, emerging problems include off-site effects and mosaicism (169).

## Intravital Imaging of Neuroinflammation

A great variety of reporters and probes are currently available to investigate neuroinflammation at the cellular level (170–173). Cellular events of interest include the migration and phagocytic activity of microglia, the infiltration of peripheral immune cells, as well as the production of secreted factors, metabolism, and viability of neural cells. Intravital imaging is useful to study the pathophysiology of neuroinflammation in a non-invasive manner, but an important limitation is the scattering and absorbance of light entering biological tissues. The availability of strong reporters and powerful imaging modalities have allowed for better detection and facilitated the generation of quantitative data from investigated signals while minimizing autofluorescence. The natural fluorescence of different tissues can mask signals from fluorescent probes. Lipofuscin, which can be excited anywhere in the range of 360–647 nm, is commonly found in neurons and glia cells, and increases with animal age. The imaging of green fluorescent protein, one of the most common and popular fluorescent labels, can also be hindered by a subset of green autofluorescent cells in the rat cortex and hippocampus (174). In tissue sections, the risk of false positives can be reduced by using autofluorescence quenchers, such as copper sulfate (175). The choice of fluorophores emitting in the near-infrared spectrum can be made to avoid this issue. Imaging of structural and functional changes in the living brain can be performed using open-skull preparations, where a small window in the skull is protected by a glass coverslip. Following the implantation of the cranial window, a recovery time is necessary to avoid inflammation caused by the surgery (176, 177). However, long-term imaging using the open-skull technique can be obscured by bone re-growth and the thickening of the meninges (178). Imaging of the cortex using the thinned-skull cranial window technique is useful when longer intervals are needed in between imaging sessions. However, repeated imaging requires the re-thinning of the skull, which has to be carefully monitored to avoid cortical trauma and inflammation (179, 180). For both imaging techniques, two-photon microscopy in the near infrared region is suitable to avoid photobleaching and photodamage.

**Figure 5:**
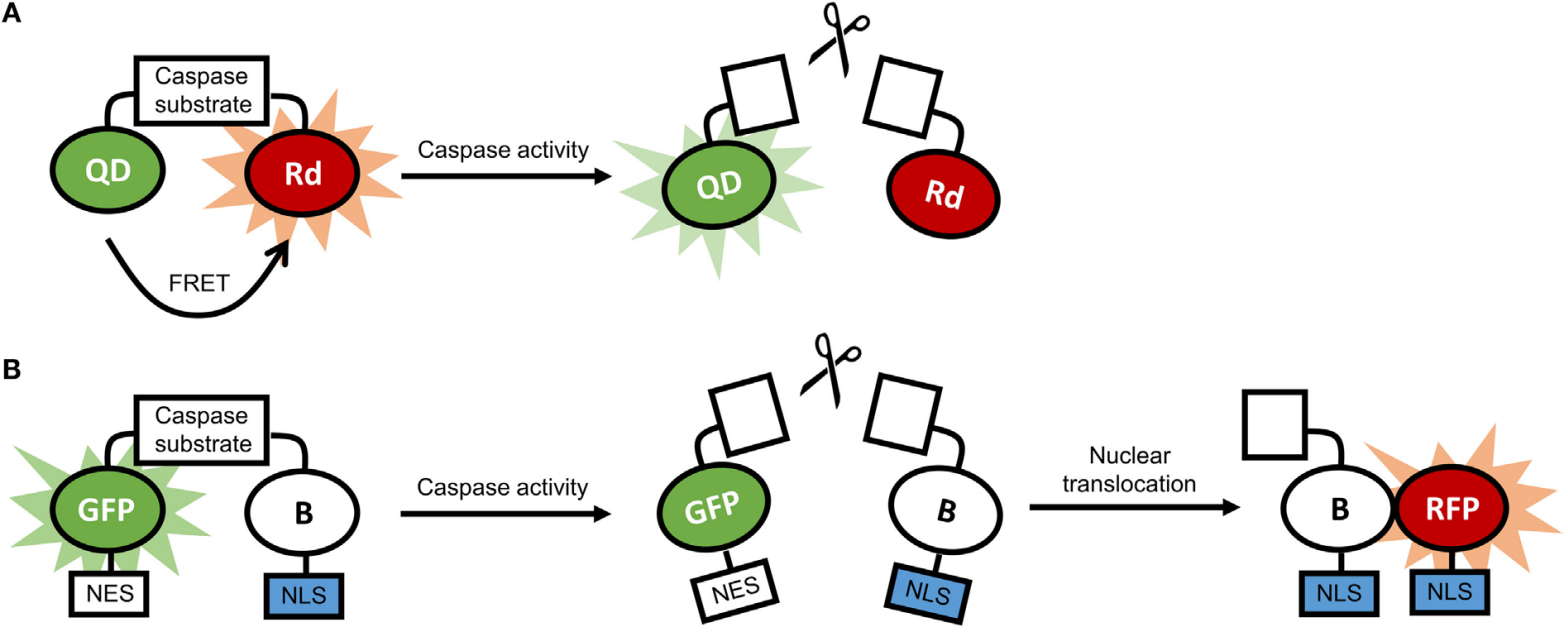
**(A)** Schematic representation of a quantum dot-based sensor for caspase activity. In the absence of caspase activity, there is fluorescence resonance energy transfer (FRET) between the quantum dot (QD) and the rhodamine molecule (Rd), and the fluorescence of the QD is quenched. In the presence of caspase activity, FRET is disrupted, and the QD is fluorescent. **(B)** Schematic representation of a ratiometric biosensor for caspase activity. In the absence of caspase activity, the dimerization-dependent green fluorescent protein (GFP) is dimerized with the partner protein B and is retained in the cytoplasm through a nuclear exclusion signal (NES). In the presence of caspase activity, the dimerization is disrupted, and B translocated to the nucleus using a nuclear localization signal (NLS), and associates with the dimerization-dependent red fluorescent protein (RFP). As a result, green fluorescence in the cytoplasm fades, and red fluorescence in the nucleus increases.

Transgenic animals expressing luciferase in glia cells have been employed to track and image processes in neuroinflammation at the cellular level (181–183). Our studies have shown marked activation of microglia, pro-inflammatory caspases, and astrocytes by nanocrystals (184–186). Data from these studies showed that stable nanocrystals injected directly into the parenchyma of mice induced transient astrocyte activation, suggesting that only nanocrystals adequately coated with polyethylene glycol (PEG) are suitable nanotechnological tools. Glia cells were also activated by gold nanoparticles, depending on the nanoparticles’ morphology (10). Activation of glia cells is often accompanied by the activation of inflammatory caspases and caspases implicated in apoptosis (187). Nanosensors for caspases have been developed, and examples of constructs for these sensors are illustrated in Figure 5 (170, 188).

A whole palette of fluorescent proteins, mostly mutant derivatives of the jellyfish’s green fluorescent protein, have also been employed to “illuminate” the brain. The use of cell-type-specific fluorescent labels allowed to map brain structures and to distinguish different cell populations with greater accuracy. High-resolution pictures have been recorded in recent years, and unprecedented 3D images and videos have been produced from fluorescently labeled brain tissues (189, 190). Although the quality of these imaging techniques remains variable and is dependent on the success of the genetic probes and the available imaging modalities, these techniques have been instrumental in understanding structural and functional aspects of the CNS – including glia–neuron interactions. Optogenetics have also been used to study light-responsive channels and other proteins in neural cells (191–193). For instance, the selective expression of channelrhodopsin-2, a light-responsive membrane channel, has been employed to study calcium signaling in astrocytes *in vitro* and *in vivo* (194). Optogenetic tools could, thus, be used to reveal the contribution of microglia in neuroinflammatory processes (195). Although optogenetics has generated valuable information on macromolecules in cells, this approach cannot be applied to investigate small molecules, such as phospholipids. More recently, the approach of optolipidomics was used to study the processing of mitochondria-specific cardiolipins in apoptosis (196). Mitochondrial functions are often impaired in inflammatory processes, and the combination of optogenetics and optolipidomics could provide complementary information on underlying intricacies in neuroinflammation.

## Conclusion

Neuroinflammation is considered a significant contributor in many neurodegenerative diseases. Microglia are the immune cells of the CNS, and are modulated by numerous factors, including alimentary products and the gut microbiome. Nanoparticulates have emerged as a new group of “xenobiotics” that must be thoroughly characterized prior to investigating their immunomodulatory effects in the CNS and elsewhere. Nanotechnology offers a wide selection of shape- and size-tunable probes, ranging from quantum dots to fluorescently labeled polymeric constructs (163). Nanoprobes can be brighter and more stable than genetic probes, and designed to “activate” in response to a particular stimuli, such as light or acidic pH. However, nanotechnological probes are often large and cannot reach desirable intracellular locations. In addition, these probes are complex and relatively little is known about their stability *in vivo*, as well as their pharmacokinetics and pharmacodynamics (186, 187). It is well established that the biological identity of a nanoparticle is distinct from its well-defined chemical identity. Serum protein binding, sensitivity to pH, and clearance rates are all factors affecting the immunogenicity and fate of a nanoparticle *in vivo* (146, 147). On the other hand, nanoparticle-induced immune responses can be exploited for improving vaccine efficiency and boost the immune system in pathologies with weakened immune responsiveness (197–199). Diverse fluorescent nanostructures can provide tools for the tracking and imaging of complex networks in different cell types in a spatio-temporal manner. The combined use of nanotechnological tools and advanced intravital imaging techniques can, thus, provide unprecedented insight into the mechanisms of neuroinflammation. Exciting data related to brain abnormalities implicating glial cells come from gene editing techniques, such as CRISPR/Cas9 (168). Animal studies exploiting these approaches in mice models of neurodegenerative diseases will help to reveal intricacies in neural circuitries under physiological conditions and mechanisms involved in multifactorial diseases associated with neuroinflammation.

## Author Contributions

DM outlined, co-wrote, and revised the manuscript. IZ drafted and finalized the figures and table, and co-authored the text. Both authors read and approved of the final version of the manuscript.

## Conflict of Interest Statement

The authors declare that the research was conducted in the absence of any commercial or financial relationships that could be construed as a potential conflict of interest.
